# A Scoping Review of Sport National Concussion Guidelines in Squash

**DOI:** 10.3390/sports13090325

**Published:** 2025-09-12

**Authors:** Nina Mangan, Neil Heron

**Affiliations:** 1 Royal Sussex County Hospital, Brighton BN2 5BE, UK; 2 Centre for Public Health, Queen’s University Belfast, Belfast BT7 1NN, UK; n.heron@qub.ac.uk

**Keywords:** squash, concussion, guidelines, Amsterdam Statement

## Abstract

Squash is a commonly played racquet sport in which players are at risk of concussion injuries. This review aims to identify and assess the squash concussion guidelines in top squash countries. **Design:** Scoping review. **Method:** This review follows the framework laid out by Arksey and O’Malley and later advanced by Levac et al. This review adheres to the PRISMA-ScR checklist. Eligibility criteria included countries with either a female or male player in the World Squash Federation Top 50 World Rankings in June 2025. This produced a list of twenty-one countries, and seven concussion guidelines were eligible for review. **Results:** Twenty-one countries matched the inclusion criteria. Canada is the only country identified with a squash-specific concussion guideline. Seven countries had national concussion guidance, and fourteen countries had no national concussion guidance. **Conclusions:** There is a lack of squash-specific concussion guidelines. The World Squash Federation and national squash organisations should produce squash-specific concussion guidelines that are in line with the Amsterdam Statement and their own respective country’s national guidelines. The World Squash Federation should specifically reference concussion in their rules and should strongly consider updating their self-inflicted injury time rules to allow for the suspension of play for up to fifteen minutes if there is a suspected head injury.

## 1. Introduction

### 1.1. What Is Sports Related Concussion (SRC) and Why Is It Important?

SRC is a traumatic brain injury that is defined as a complex pathophysiological process affecting the brain, induced by biomechanical forces with several common features that help define its nature [1]. Symptoms and signs of concussion include loss of consciousness, confusion, increased irritability, behaviour changes, short term memory loss, headache, vertigo, nausea, vomiting, visual changes and seizures [2].

There is no one diagnostic test for concussion and it can therefore be notoriously difficult to diagnose [3]. Imaging tests such as magnetic resonance imagining (MRI) and computed tomography (CT) can often appear normal, and the diagnosis can only be made clinically [4]. Therefore, having a standardised and evidence-based approach to the assessment of concussion is imperative.

The consequences of missing a diagnosis of concussion are severe as misdiagnosis or faulty management can ‘lead to major disability or death’ [4]. If an athlete has suffered a head injury and returns to sport too early, they are at risk of sustaining a second head injury which may lead to second-impact syndrome [5]. This is a rare condition that likely occurs due to the brain being unable to recover from the initial head injury and can lead to death of the athlete. Additionally, accurately making a diagnosis of concussion is important as repeated instances of concussion can potentially lead to delayed post-traumatic brain degeneration, leading to conditions such as dementia, Alzheimer’s disease and Parkinson diseases [6]. Repeated concussions are also a risk factor for mental health problems such as depression in later life [7].

### 1.2. The Amsterdam Statement

In 2022, the Concussion in Sport Group released a consensus statement on SRC [8]. This document builds upon its five previous statements from 2001, 2004, 2008, 2012 and 2017, respectively [9,10,11,12,13]. The consensus statements released by the Concussion in Sport Group are produced by a panel of international experts, who meet every three to five years to review the latest research on SRC and subsequently release advice and guidance.

The Amsterdam Statement focuses on the thirteen ‘Rs’ of SRC; recognise, reduce, remove, refer, re-evaluate, rest, rehabilitate, recover, return-to-learn/return-to-sport, reconsider, residual effects, retire and refine.

### 1.3. Are Squash Players at Risk of Concussion?

Squash is a high intensity racquetball sport normally played to the best of five games [14]. The majority of squash injuries are musculoskeletal and soft tissue injuries. However, a significant proportion of injuries are head and eye related. The incidence rates of eye and head injuries in squash range from 5.2 to 33.3 injuries per 100,000 playing sessions [15]. The incidence of concussion in squash is unclear; a literature search on Pubmed with ‘squash’, ‘concussion’, ‘incidence’ and ‘rate’ does not yield a reliable result. Direct comparison to other sports is therefore limited and the exact incidence of concussion in squash is an area for future research. However, it is clear, due to the nature of the sport, that squash players are at risk of concussion. Squash is a fast-paced racquetball game played in close quarters. Players are therefore at risk of falling and hitting their heads against the court walls and/or being hit with equipment. In recent years, there have been several athletes who have had to withdraw from matches due to suspected on court concussion injuries, such as Lucas Serme in the 2022 US Open, and Lionel Cardenas in the 2025 PSA World Championships [16,17]. As highlighted earlier, the consequences of a missed concussion diagnosis are severe, and clear guidance is therefore needed.

### 1.4. Who Regulates Squash?

There are two main regulating bodies in squash. The professional tour is governed by the Professional Squash Association (PSA). The World Squash Federation (WSF) outlines the rules of squash, and they include a section on timeouts and suspension of play due to injury. However, they make **no reference** to concussion. Instead, the WSF focuses on the categorisation of injury as self-inflicted, contributed or opponent-inflicted [14]. At an individual country level, each country has a national squash governing body that oversees squash at a national level. Since the publishing of the Berlin Statement in 2017, there has been widespread implementation of sport specific concussion guidelines, with individual sports developing specific concussion guidelines to use within their sport [18,19,20,21,22]. However, there is no literature assessing the adequacy of squash-specific concussion guidelines. A search on Pubmed with the terms ‘squash’ ‘concussion’ and ‘guideline’ produces no results.

### 1.5. Aims

This study aims to identify the squash concussion guidelines in top squash countries. Subsequently this study aims to assess if these guidelines are compliant with the recommendations set out in the Amsterdam Statement.

## 2. Materials and Methods

A scoping review was selected as the study method. This study design was selected as there is currently no overview in the literature regarding squash-specific concussion policy. This article follows the framework proposed by Arksey and O’Malley in 2005, and later advanced by Levac et al. in 2010 [23,24]. Additionally, this study adheres to the PRISMA-ScR (Preferred Reporting Items for Systematic reviews and Meta-Analyses extension for Scoping Reviews) checklist [25]. The completed PRISMA checklist is provided in the Supplementary Table S1.

### 2.1. Stage 1: Identify the Research Question(s)

1. Which national squash governing bodies have published concussion guidelines appropriate for use in squash?
2. If a country does not have a squash-specific concussion guideline, does it have a national or grassroots sport concussion guideline that is appropriate for use in squash?
3. Do the current guidelines follow the Amsterdam Statement recommendations?

### 2.2. Stage 2: Identifying Relevant Studies

This study focused on countries that have a ranked female or male player in the Top 50 WSF rankings as of 2 June 2025 [26,27].

### 2.3. Stage 3: Study Selection

The database ‘Pubmed’ was searched from 2004 to 2005 with the keywords ‘squash’, ‘concussion’ and ‘guideline.’ This produced no results.

A hand selection process was therefore performed. A list of the national squash governing body of each country was obtained from the WSF website [28]. Official national squash organisation websites were accessed, and their published public materials reviewed. If a squash organisation did not have a concussion policy, the official national sport and public health website of that country were accessed, and their published public materials reviewed to assess if they had national sporting concussion guidelines. This produced a further nine records. Grey literature was not routinely included.

Records were assessed for eligibility and duplicates removed. This finally produced seven records eligible for this study. See Appendix A for PRISMA study selection flowchart.

### 2.4. Stage 4 and 5: Charting the Data and Collating, Summarising, and Reporting Results

The data obtained from the relevant materials were displayed in tables using Microsoft Word. The data extracted included author, title, year, publisher and concussion-related guidance. Data was extracted by Nina Mangan.

## 3. Results

### 3.1. Individual Country’s Guidelines

This study focused on countries that had a female or male player in the Top 50 WSF rankings as of 2 June 2025 [26,27]. This produced a list of twenty-one countries; Belgium, Canada, Colombia, Egypt, England, France, Germany, Hong Kong, Hungary, India, Japan, Malaysia, Mexico, New Zealand, Peru, Qatar, Scotland, Spain, Switzerland, USA and Wales. See Table 1. 

### 3.2. Recognition

The Amsterdam Statement highlights that some of the greatest challenges in the management of SRC are the initial diagnosis and assessment [8]. A thorough sideline assessment of a player with suspected concussion is therefore critical. The Amsterdam Statement highlights that standard orientation questions are insufficient in diagnosing concussion. In order to correctly identify cases of concussion, the Amsterdam Statement therefore recommends the use of screening tools, in particular the Concussion Recognition Tool-6 (CRT6), the Sport Concussion Assessment Tool-6 (SCAT6) and Child SCAT6. A comparison on the different guidance on the initial assessment of concussion can be found in Table 2. 

### 3.3. Rest and Return to Play (RTP)

The Amsterdam Statement acknowledges that there is no clear consensus in the literature regarding the appropriate duration of rest after a player has SRC or a suspected SRC [8]. The Amsterdam Statement recommends that a relative rest period of twenty-four to forty-eight hours is appropriate, followed by a gradual return to sporting activities. These stages include aerobic exercise, individual sport-specific exercise, non-contact training drills, full contact practice, and finally return to sport. Each stage of RTP should last a minimum of twenty-four hours. See Table 3 for a comparison on the guidance surrounding rest and return to play.

## 4. Discussion

### 4.1. Overview

Twenty-one countries were identified as a having a female or male player in the WSF Top 50 World Rankings as of 2 June 2025. Canada is the only country with a squash-specific concussion guideline, while six countries have national SRC guidelines. Fourteen countries have neither a squash-specific concussion guideline nor national SRC guideline.

### 4.2. Individual Country’s Guidelines

Canada is the only country identified that has a squash-specific concussion policy. In 2015, the Public Health Agency of Canada launched a Concussion Protocol Harmonization Project [36]. This initiative aimed to have a national and cohesive approach to SRC. Canada’s first national SRC guideline was published in 2017, with an updated second edition published in 2024 [30]. As part of this process, forty-nine national sports organisations were engaged and a standardised approach to concussion implemented. In 2019, Squash Canada launched their own squash-specific concussion policy as part of this wider project [29]. A homogenous approach to concussion helps ensure that there is clear guidance for the assessment of players with a suspected SRC and clear advice regarding their subsequent management. It also allows for easier research into the diagnosis, assessment and management of concussion.

Six countries (England, New Zealand, Qatar, Scotland, USA and Wales) have no squash-specific concussion guidelines but do have national guidelines. However, in all these countries, excluding Qatar, their guidance is intended for players at a community level rather than professional sports players. A standardised approach to concussion in grassroots sports is important; however, it is also necessary to develop squash-specific concussion guidelines to ensure a consistent approach in the elite, professional game, where closer monitoring and more intensive treatment can often be instigated compared to the community level.

At elite-level sport, adherence to injury time rules is more tightly regulated than at a grassroots level. As noted earlier, the rules of squash are regulated by the WSF. There is no mention of concussion in the WSF rules [14]. Instead, the focus on injury is subdivided into self-inflicted, contributed and opponent inflicted. If the injury is self-inflicted, the player has three minutes to recover and resume. If they are unable to do this, they must concede and take the game interval. This is an exceptionally short time period to rule out concussion, especially given there is currently no definitive guidance for concussion. In comparison, World Rugby has introduced a head injury assessment clause to their rules [37]. In rugby, if a player has a suspected concussion, a temporary replacement is allowed for a twelve-minute period (even if all substitutes have been used) to allow for the safe and thorough assessment of concussion. The Amsterdam Statement highlights that using a multimodal screen to evaluate potential concussion takes at least ten to fifteen minutes [8]. The WSF should strongly consider updating their self-inflicted injury time rules to allow for the suspension of play for up to fifteen minutes if there is a suspected head/concussion injury.

The need for distinct guidelines for elite squash players is also needed due to the additional resources available to professional athletes. At elite sporting matches, there will likely be trained healthcare professionals who can make use of evidence-based concussion sideline assessment tools such as SCAT6. A guideline that advocates for the use of such assessments is needed.

Other sports have developed and published specific concussion guidance. For example, in the UK, sports such as American football, basketball, cricket, Gaelic, gymnastics, hockey, eventing, ice hockey, judo, netball, rugby and football have developed their own specific concussion protocols [38]. National squash bodies should therefore develop their own concussion guidelines.

Fourteen countries (Belgium, Colombia, Egypt, France, Germany, Hong Kong, Hungary, India, Japan, Malaysia, Mexico, Peru, Spain and Switzerland) have no concussion squash-specific guidance or national guidance. Squash players in these countries are not covered by any clear concussion guidance, and this should therefore be urgently addressed.

### 4.3. Recognition of Concussion

In Canada’s guidelines and countries with national guidance, immediate removal of the player with suspected concussion is featured in all guidance. Additionally, a detailed list of the signs and symptoms of concussion are included in all available guidance. These are welcome findings.

However, only two countries (Canada and Qatar) follow the Amsterdam Statement recommendation of using concussion specific screening tools. The other available national guidelines are focused on recommendations for non-elite sports. As noted earlier, concussion is notoriously a difficult diagnosis to make. There is no one specific test for concussion and the diagnosis is made clinically [3,4]. At an elite level sport, where there are trained healthcare professionals available, the use of screening tools such as SCAT6 should be standard practice and advocated for in concussion policy, with these tools being free, non-invasive and effective screening tools [39]. The consequences of not using screening tools are substantial. As noted earlier, a missed diagnosis of concussion can lead to second impact syndrome, disability and even death of the athlete [4].

### 4.4. Rest and Return to Play

The majority of guidelines recommend an initial recovery period of 24–48 h, which is in line with the Amsterdam Statement recommendations. All guidance included some form of return to play (RTP); however, the recommendations varied in nature.

Squash Canada has outlined a return-to-school strategy as well as a squash-specific return-to-sport strategy. New Zealand’s national guidance includes a graduated return to education/work and sport protocol. Qatar has provided a return-to-learn and return-to-sport pathway. In the US, the CDC has released a 6-step Return to Play Progression, but this is focused on children and does not include guidance on return to work or high-level sport. The UK government guidance includes a graduated return-to-activity (education/work) and sport programme. The Scottish guidance also contains a graduated return-to-activity (education/work) and sport programme.

Squash Canada and the US guidance do not give a specific timeframe for RTP. Qatar’s guideline advises that one week post injury is the earliest point at which return to sport can be achieved. New Zealand and the UK (including Scotland) specifically note that return to sport that poses risk of injury can only be undertaken when fourteen days symptom free and return to competition can only be undertaken at twenty-one days at the community level. The updated guidance from the Amsterdam Statement highlights that players are safely able to return to full contact training at six days and return to sport at seven days. Indeed, delaying return to sport for players could have unintended consequences such as decreased physical fitness and a psychological impact. The consequences of a delayed return to sport will likely affect professional squash players more and has the potential to lead to ranking loss, income loss or interrupted competitive momentum.

### 4.5. Implications of Research

The Amsterdam Statement highlights the importance of sport-specific concussion guidelines. This is especially important in the prevention of concussion. The Amsterdam Statement highlights ice hockey as an example where a change in policy has helped to reduce concussion. Ice hockey policies have changed and ‘body checking’ is no longer allowed in child or adolescent ice hockey [8]. This had reduced concussion rates by 58% [40]. Squash-specific concussion policies should reflect on ways that concussion in squash could potentially be prevented.

The WSF should specifically reference and provide guidance for concussion in the injury time section of their rules. The WSF should strongly consider amending the self-inflicted injury time section of their rules to allow suspension of play for up to fifteen minutes if there is a head injury.

The WSF and national squash organisations should produce squash-specific guidelines for players that are in line with the Amsterdam Statement recommendations. These guidelines should reference the ‘13 Rs’ of SRC [8]. In particular, these guidelines should provide guidance on the recognition of concussion, advocate for the immediate removal of a player with suspected concussion, recommend the use of sideline assessment tools such as CRT6, SCAT6 and child SCAT6, and provide clear guidance on return to play.

### 4.6. Strengths and Limitations

This is the first systematic scoping review of concussion guidelines in the sport of squash. This review analysed the squash concussion policies of countries that had a female or male player in the Top 50 WSF rankings. This review therefore provided a good oversight of the concussion policies in top squash playing countries, although it does acknowledge that there are other countries that play squash, and their squash concussion policies were not assessed as part of this project. Further research should expand to all WSF members to provide a more global view of national concussion policies in squash. There is limited information in the literature regarding the exact incidence of concussion in squash; this is an area for future research.

## 5. Conclusions

In the world’s top squash countries, there is a great variation in SRC policies. Canada is the only country with a specific squash concussion policy. In the remaining countries, six had national SRC guidance for grassroots sport, while fourteen had no national SRC guidance.

All guidelines available provided a comprehensive list of the signs and symptoms of concussion, and all guidelines emphasised the importance of immediate removal from play if players had a suspected diagnosis of concussion. Only two countries recommended the use of concussion specific screening tools, and there were differences in the recommended RTP pathways.

The WSF should convene an expert panel to draft squash-specific concussion guidelines within the next two years. These guidelines should be in line with the recommendations from the Amsterdam Statement. The WSF should make specific reference to concussion in the injury time section of their rules. The WSF should amend the self-inflicted injury time section of their rules to allow for the suspension of play for up to fifteen minutes if there is a suspected head injury. National squash organisations should produce SRC guidelines that follow the Amsterdam Statement recommendations, the WSF recommendations and their own respective country’s national guidelines. These guidelines should advocate for the use of evidence-based sideline assessment tools, the immediate removal of players with suspected concussion, and clear guidance on RTP.

## Figures and Tables

**Table 1:** Concussion policies of top squash countries.

Country	Governing Body	Squash-Specific Concussion Policy	National Sports Related Concussion Policy
Belgium	Belgian Squash Federation	No	No
Canada	Squash Canada	Yes—Squash Canada Concussion Protocol [29]	Yes—Canadian Guideline on Concussion in Sport [30]
Colombia	Colombian Squash Federation	No	No
Egypt	Egyptian Squash Federation	No	No
England	England Squash	No	Yes—UK Concussion Guidelines for Non-Elite (Grassroots) Sport November 2024 [31]
France	French Squash Federation	No	No
Germany	German Squash Association	No	No
Hong Kong	Squash Association of Hong Kong, China	No	No
Hungary	Hungarian Squash Association	No	No
India	Squash Rackets Federation of India	No	No
Japan	Japan Squash Association	No	No
Malaysia	Squash Racquets Association of Malaysia (SRAM)	No	No
Mexico	Mexico Squash Association	No	No
New Zealand	Squash New Zealand	No	Yes—Sport Concussion in New Zealand: National Guidelines [32]
Peru	Peruvian Squash Federation	No	No
Qatar	Qatar Squash Federation	No	Yes—The Aspetar Sport Related Concussion Programme [33]
Scotland	Scottish Squash	No	Yes—If in doubt, sit them out. Scottish Sports Concussion Guidance: grassroots sport and general public [34]
Spain	Spanish Squash Federation	No	No
Switzerland	Swiss Squash	No	No
United States	US Squash	No	Guidance available on the Centers for Disease Control and Prevention website [35]
Wales	Squash Wales	No	UK Concussion Guidelines for Non-Elite (Grassroots) Sport November 2024 [31]

Note: the Welsh Government document ‘Welsh Government guidance on concussion for school and community sport up to age 19’ was not included in this analysis as it focuses on concussion in players up until the age of 19.

**Table 2:** Guidance on initial assessment of concussion.

Country	Sideline Assessment and Recommendation of Screening Tools	List of Signs and Symptoms of Concussion	Immediate Removal of Player with Suspected Concussion
Belgium	N/A *	N/A	N/A
Canada	Yes—recommends CRT5, SCAT5 and Child SCAT5	Yes	Yes
Colombia	N/A	N/A	N/A
Egypt	N/A	N/A	N/A
England	No	Yes	Yes
France	N/A	N/A	N/A
Germany	N/A	N/A	N/A
Hong Kong	N/A	N/A	N/A
Hungary	N/A	N/A	N/A
India	N/A	N/A	N/A
Japan	N/A	N/A	N/A
Malaysia	N/A	N/A	N/A
Mexico	N/A	N/A	N/A
New Zealand	No	Yes	Yes
Peru	N/A	N/A	N/A
Qatar	Yes—recommends CRT6, SCAT6 and SCOAT6	Yes	Yes
Scotland	No	Yes	Yes
Spain	N/A	N/A	N/A
Switzerland	N/A	N/A	N/A
United States	No	Yes	Yes
Wales	No	Yes	Yes

* Not applicable.

**Table 3:** Guidance of rest and return to play.

Country	Initial Period of Relative Rest (hours)	Guidance on Return to Play	Notes
Belgium	N/A **	N/A	N/A
Canada	24–48	Yes	Return-to-School Strategy Squash-Specific Return-to-Sport Strategy
Colombia	N/A	N/A	N/A
Egypt	N/A	N/A	N/A
England	24	Yes	Graduated return to activity (education/work) and sport programme
France	N/A	N/A	N/A
Germany	N/A	N/A	N/A
Hong Kong	N/A	N/A	N/A
Hungary	N/A	N/A	N/A
India	N/A	N/A	N/A
Japan	N/A	N/A	N/A
Malaysia	N/A	N/A	N/A
Mexico	N/A	N/A	N/A
New Zealand	24–48	Yes	Includes a return to work/sport guide
Peru	N/A	N/A	N/A
Qatar	Yes		Return to sport/learning guidance
Scotland	24–48	Yes	Return to work/play guidance
Spain	N/A	N/A	N/A
Switzerland	N/A	N/A	N/A
United States	Not explicitly stated	Yes	6-step return to play progression Focus on children
Wales	24	Yes	Graduated return to activity (education/work) and sport programme

** Not applicable.

## Supplementary Materials

The following supporting information can be downloaded at: https://www.mdpi.com/article/10.3390/sports13090325/s1, Table S1: Preferred Reporting Items for Systematic reviews and Meta-Analyses extension for Scoping Reviews (PRISMA-ScR) Checklist.

## References

[1] McCrory P., Feddermann-Demont N., Dvořák J., Cassidy J.D., McIntosh A., Vos P.E., Echemendia R.J., Meeuwisse W., Tarnutzer A.A. (2017). What is the definition of sports-related concussion: A systematic review. Br. J. Sports Med..

[2] NHS Inform (2025). Concussion. NHS 24:UK. https://www.nhsinform.scot/illnesses-and-conditions/injuries/head-and-neck-injuries/concussion/.

[3] Daly E., Pearce A.J., Finnegan E., Cooney C., McDonagh M., Scully G., McCann M., Doherty R., White A., Phelan S. (2022). An assessment of current concussion identification and diagnosis methods in sports settings: A systematic review. BMC Sports Sci. Med. Rehabil..

[4] Tator C.H. (2013). Concussions and their consequences: Current diagnosis, management and prevention. Can. Med Assoc. J..

[5] May T., Foris L.A., Donnally C.J. (2023). Second Impact Syndrome. StatPearls.

[6] McLendon L.A., Kralik S.F., Grayson P.A., Golomb M.R. (2016). The Controversial Second Impact Syndrome: A Review of the Literature. Pediatr. Neurol..

[7] Manley G., Gardner A.J., Schneider K.J., Guskiewicz K.M., Bailes J., Cantu R.C., Castellani R.J., Turner M., Jordan B.D., Randolph C. (2017). A systematic review of potential long-term effects of sport-related concussion. Br. J. Sports Med..

[8] Patricios J.S., Schneider K.J., Dvorak J., Ahmed O.H., Blauwet C., Cantu R.C., Davis G.A., Echemendia R.J., Makdissi M., McNamee M. (2023). Consensus statement on concussion in sport: The 6th International Conference on Concussion in Sport–Amsterdam, October 2022. Br. J. Sports Med..

[9] McCrory P., Meeuwisse W., Dvořák J., Aubry M., Bailes J., Broglio S., Cantu R.C., Cassidy D., Echemendia R.J., Castellani R.J. (2017). Consensus statement on concussion in sport: The 5th International Conference on Concussion in Sport held in Berlin, October 2016. Br. J. Sports Med..

[10] Aubry M., Cantu R., Dvorak J., Graf-Baumann T., Johnston K., Kelly J., Lovell M., McCrory P., Meeuwisse W., Schamasch P. (2002). Summary and agreement statement of the First International Conference on Concussion in Sport, Vienna 2001. Physician Sportsmed..

[11] McCrory P., Johnston K., Meeuwisse W., Aubry M., Cantu R., Dvorak J., Graf-Baumann T., Kelly J., Lovell M., Schamasch P. (2005). Summary and agreement statement of the 2nd International Conference on Concussion in Sport, Prague 2004. Br. J. Sports Med..

[12] McCrory P., Meeuwisse W., Johnston K., Dvorak J., Aubry M., Molloy M., Cantu R. (2009). Consensus statement on concussion in sport: The 3rd International Conference on Concussion in Sport held in Zurich, November 2008. S. Afr. J. Sports Med..

[13] McCrory P., Meeuwisse W.H., Aubry M., Cantu B., Dvořák J., Echemendia R.J., Engebretsen L., Johnston K., Kutcher J.S., Raftery M. (2013). Consensus statement on concussion in sport: The 4th International Conference on Concussion in Sport held in Zurich, November 2012. Br. J. Sports Med..

[14] World Squash Federation (2024). Rules of Singles Squash 2024.

[15] Finch C., Eime R. (2001). The epidemiology of squash injury. Int. Sport. J..

[16] PSA Squash Tour Lucas Serme Withdraws US Open Third Round (10 October 2022). https://www.psasquashtour.com/news/lucas-serme-withdraws-us-open-third-round/.

[17] PSA Squash Tour Cardenas Pulls Out PSA World Championship Third Round (12 May 2025). https://www.psasquashtour.com/featured-news/cardenas-pulls-out-psa-world-championship-third-round/.

[18] Davis G.A., Makdissi M., Bloomfield P., Clifton P., Cowie C., Echemendia R., Falvey E.C., Fuller G.W., Green G.A., Harcourt P. (2020). Concussion Guidelines in National and International Professional and Elite Sports. Neurosurgery. Neurosurgery.

[19] The Football Association (2023). If in Doubt, Sit Them Out: English FA Concussion Guidelines.

[20] Rugby Football Union (2023). Headcase: Extended Guidelines.

[21] British Cycling (2023). Concussion Guidelines.

[22] England Hockey (2018). Concussion Policy.

[23] Arksey H., O’Malley L. (2005). Scoping studies: Towards a methodological framework. Int. J. Soc. Res. Methodol..

[24] Levac D., Colquhoun H., O’Brien K.K. (2010). Scoping studies: Advancing the methodology. Implement. Sci..

[25] Tricco A., Zarin L.E., O’Brien K., Colquhoun H., Levac D. (2018). Preferred Reporting Items for Systematic reviews and Meta-Analyses extension for Scoping Reviews (PRISMA-ScR) Checklist SECTION. Ann. Intern. Med..

[26] Squash Info Women’s PSA World Squash Rankings. https://www.squashinfo.com/rankings/women.

[27] Squash Info Men’s PSA World Squash Rankings. https://www.squashinfo.com/rankings/men.

[28] World Squash Federation WSF Information: Member Nations. https://www.worldsquash.org/wsf-information/member-nations-2-2/.

[29] Squash Canada (2019). Squash Canada Concussion Protocol.

[30] Parachute Canada (2024). Canadian Guideline on Concussion in Sport.

[31] Sport and Recreation Alliance (2024). UK Concussion Guidelines for Grassroots.

[32] ACC (2024). Sport Concussion in New Zealand: National Guidelines.

[33] Aspetar Orthopaedic and Sports Medicine Hospital Sports Concussion. https://www.aspetar.com/en/patient-information/common-sports-injuries/head/sports-concussion.

[34] Sportscotland (2024). If in Doubt, Sit Them Out. Scottish Sports Concussion Guidance: Grassroots Sport and General Public.

[35] Centers for Disease Control and Prevention (n.d.). Heads Up: Concussion and Head Injury Awareness.

[36] Parachute Canada (2024). Concussion Collection: Concussion Harmonization Project.

[37] World Rugby (2025). Laws of the Game Rugby Union.

[38] Scullion E., Heron N. (2022). A Scoping Review of Concussion Guidelines in Amateur Sports in the United Kingdom. Int. J. Environ. Res. Public Health.

[39] Echemendia R.J., Burma J.S., Bruce J.M., Davis G.A., Giza C.C., Guskiewicz K.M., Naidu D., Black A.M., Broglio S., Kemp S. (2023). Acute evaluation of sport-related concussion and implications for the Sport Concussion Assessment Tool (SCAT6) for adults, adolescents and children: A systematic review. Br. J. Sports Med..

[40] Eliason P.H., Galarneau J.-M., Kolstad A.T., Pankow M.P., West S.W., Bailey S., Miutz L., Black A.M., Broglio S.P., Davis G.A. (2023). Prevention strategies and modifiable risk factors for sport-related concussions and head impacts: A systematic review and meta-analysis. Br. J. Sports Med..

